# Impacts of pr-10a Overexpression at the Molecular and the Phenotypic Level

**DOI:** 10.3390/ijms140715141

**Published:** 2013-07-22

**Authors:** Lea A. I. Vaas, Maja Marheine, Johannes Sikorski, Markus Göker, Heinz-Martin Schumacher

**Affiliations:** 1 DSMZ–German Collection for Microorganisms and Cell Cultures, Inhoffenstr. 7b, 38124 Braunschweig, Germany; E-Mails: mmh@dsmz.de (M.M.); johannes.sikorski@dsmz.de (J.S.); markus.goeker@dsmz.de (M.G.); mas@dsmz.de (H.-M.S.); 2 Centraalbureau voor Schimmelcultures–Fungal Biodiversity Center, Uppsalalaan 8, 3584CT Utrecht, The Netherlands

**Keywords:** pathogenesis related protein 10a, cell-respiration, TTC, *Solanum tuberosum* cv. Désirée

## Abstract

Biotechnological approaches using genetic modifications such as homologous gene overexpression can be used to decode gene functions under well-defined circumstances. However, only the recording of the resulting phenotypes allows inferences about the impact of the modification on the organisms’ evolutionary, ecological or economic performance. We here compare a potato wild-type cell line with two genetically engineered cell cultures homologously overexpressing Pathogenesis Related Protein 10a (*pr-10a*). A detailed analysis of the relative gene-expression patterns of *pr-10a* and its regulators *sebf* and *pti4* over time provides insights into the molecular response of heterotrophic cells to distinct osmotic and salt-stress conditions. Furthermore, this system serves as an exemplar for the tracing of respiration kinetics as a faster and more sensitive alternative to the laborious and time-consuming recording of growth curves. The utility and characteristics of the resulting data type and the requirements for its appropriate analysis are figured out. It is demonstrated how this novel type of phenotypic information together with the gene-expression-data provides valuable insights into the effect of genetic modifications on the behaviour of cells on both the molecular and the macroscopic level.

## 1. Introduction

When plant cells are faced with osmotic or salt challenges, major changes in gene-expression levels are an intrinsic part of the drastic action triggering the physiological package of measures for stress response [1,2]. Because elevated PR-10a proteins are repeatedly found in salt and osmotically stressed entire plants, as well as cell cultures [3–7], a role in stress perception or signal transduction has been postulated [7].

The first reports of the elicitor-induced appearance of mRNAs of the pathogenesis-related protein 10a (PR-10a, formerly known as STH-2) dates back more than 20 years ago [8,9]. Meanwhile, diverse studies reported *pr-10a* gene-expression or protein abundance to be induced by several biotic and abiotic stressors in various plants, organs, tissues [6,10–14] and developmental stages [15,16]. A detailed analysis of the expression pattern of the *pr-10a* gene in *Solanum tuberosum* plants of cultivar Désirée revealed that no major organ exhibited constitutive expression [10]. Only the already known expression induction after infection, elicitor treatment, or, to a lower extent, after wounding could be confirmed when additional information about the magnitude of expression induction in vascular bundles, roots and leaves, as well as in stigmas, was obtained [10].

Modulating the expression of *pr-10a* by genetic engineering yielded inconsistent results (reviewed in [17]). Whereas, other studies reported enhanced salt and/or osmotic tolerance due to *pr-10a* overexpression [3,18,19], the results in the context of pathogen attack are not that easy to interpret. In potato plants, overexpressing *pr-10a* did neither lead to increased resistance against *Phytophthora infestans* nor against potato virus X [20], whereas in the legume *Medicago truncatula*, silencing of *PR-10*-like proteins increased the tolerance against infection with *Aphanomyces euteiches* [21].

Studies on the regulatory processes of *pr-10a* gene expression yielded more comprehensive results [17] and led to the description of an interplay of a repressosome and an activator complex [22]. Based on findings about the phosphorylation status of nuclear factor PBF-1 [23], and the involvement of the single-stranded DNA binding factor Why1 (formerly PBF-2; [24]) in *pr-10a* gene activation and on observations of *pr-10a* repression by the single-stranded DNA binding protein SEBF [25], it was hypothesized that the *pr-10a* gene has two different activity states. In the inactivated state, a repressosome, consisting of a heterodimeric SEBF-Pti4 complex (an ethylene-response transcription factor), occupies the silencer element of the promoter [22]. To become active, the repressosome has to be dismissed, thus allowing for the recruitment of Why1 to the upstream elicitor response element in the promoter [22,24].

Even though it is widely accepted that the gene does not encode a substantial new feature such as a ion pump [17,19], the modes of action of the PR-10a protein itself, as well as the pathways it could interfere with, are an object of active research [17,22]. Beside reports about RNA hydrolysis [26,27], the exploration of binding capacities of proteins of the PR-10 family from different plants revealed high cytokinin affinity [28,29], and other possible ligands such as fatty acids, flavonoids [28,30] or brassinosteroids [31] were postulated. Additionally, possible crosstalks with hormone-signalling pathways [32] as well as interactions with the mitogen-activated protein kinase cascades were reported [33]. Further studies reported cryoprotective activity of PR-10/Bet v 1 protein homologues in mulberry [14]. To the best of our knowledge, however, none of the described PR-10a features were observed *in vivo*, thus the actual role of PR-10a proteins in living cells remains unclear [17,34].

Since the effect of changed pr-10a-expression levels are ultimately displayed at the cellular level, as cellular traits or phenotypes are apparent in growth curves [19], the need for investigations at the phenotypic level is apparent. However, since the very first responses of plant cells to salt or osmotic stress are rapid (within minutes to hours [35]), the commonly applied, but comparably slowly progressing, growth measurements are not appropriate to determine metabolic phenotypes. Recently the principle of studying an organisms’ behaviour on a longitudinal level was augmented by the introduction of a highly automated system measuring microbial respiration over time in a highly parallelized manner, the cells being faced with a large number of distinct physiological challenges [36–38]. Although those assays are well known in plant sciences [39–41], traditional applications of this assay mainly aimed at only qualitatively determining the viability of cells (dead versus alive) [42–44].

Since the successful application of this triphenyltetrazolium chloride (TTC)-based approach was reported for eukaryotes, such as filamentous fungi [45] and mammals [46], it can be postulated that this technique would permit the determination of longitudinal respiratory phenotypes in a plant-cell system, too.

A potato wild-type cell line (*Solanum tuberosum* cv. Désirée) together with two transgenic cell lines homologously overexpressing the *pr-10a* gene, here serves as a model system. Based on a detailed longitudinal analysis regarding the relative gene-expression patterns of *pr-10a* as well as *sebf* and *pti4*, whose gene products are involved in the repressosome, the gene-expression response of heterotrophic plant cells to distinct osmotic and salt-stress conditions are exemplified. To phenotypically describe the treatments’ effects on the traditional long-term scale, osmotic and salt-stress responses were determined by measurement of cells’ growth behaviour.

Finally, to gather phenotypic information about the very first phase of the salt and osmotic stress responses, an adaptation of TTC-based viability assays for plant cell-culture systems is introduced and its usability as a fast and feasible alternative for monitoring metabolic phenotypes over time at the cellular level is demonstrated. Its technical realisation is discussed and the characteristics of the resulting data type, as well as requirements for its appropriate analysis, are figured out.

## 2. Results

### 2.1. Magnitude of pr-10a Expression Induction Is Sensitive to both the Chemical Compound and the Strength of Osmotic Pressure

The monitoring of *pr-10a* expression was accomplished by treating the cells with osmotic (0.5 M sorbitol) and salt stress (0.16 M and 0.32 M NaCl) followed by expression measurement over time, as described in detail in the experimental section. The according relative expression levels of *pr-10a* (normalized against *18S rDNA* [47]) are provided in Figure S1.

**Figure 1:**
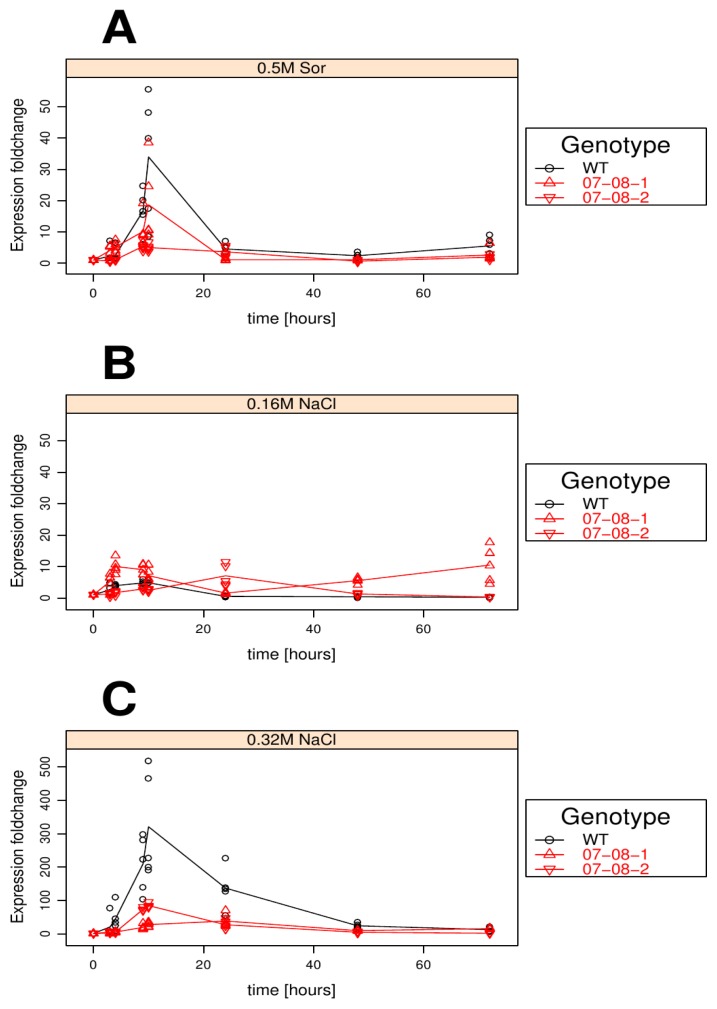
Time course of *pr-10a* gene-expression fold changes of cells treated with medium supplemented with (**A**) 0.5 M sorbitol, (**B**) 0.16 M NaCl and (**C**) 0.32 M NaCl. Measurements for all five independent samples per time point and treatment group are displayed as symbols (circles and triangles) indicating individual measurements and lines giving the averages.

In the high osmotic pressure treatments of 0.5 M sorbitol (Figure 1A) or the equiosmolal 0.32 M NaCl (Figure 1C), the pattern of gene-expression induction over time exhibited its maximum at 10 h after the start of treatment followed by a decline back to the starting levels after two days, irrespective of the genotype. In the wild-type cells, the magnitude of this gene-expression induction differed dramatically between the two osmotic treatments. Whereas the 0.5 M sorbitol treatment induced *pr-10a* gene expression to a relative fold change of about 35 (Figure 1A), the equiosmolal 0.32 mM NaCl treatment caused a much larger fold change of about 300 (Figure 1C). In contrast, both transgenic cell lines exhibited much less gene-expression induction. Genotype 07-08-1 reacted with a *pr-10a* expression fold change of around 20 to the sorbitol treatment (Figure 1A) and genotype 07-08-2 with a fold change around five.

Surprisingly, the 0.16 M NaCl treatment entailed a fold change of only five to 10 in all the genotypes, indicating only a weak *pr-10a* gene-expression induction (Figure 1B). However, genotype 07-08-1 showed a slight expression induction at the last time point after a 72 h stress treatment.

Regarding the relative levels of expression, all genotypes reacted to the mild 0.16 M NaCl and the 0.5 M sorbitol treatments with similar rates of expression induction (see Figure S1). Due to the generally higher expression level of the transgenic cell lines, they performed at different levels, but otherwise reacted like the wild-type cells throughout the observed period of time (see Figure S1). However, the 0.32 M NaCl treatment apparently entailed such a severe *pr-10a* expression induction in the wild-type cells, that they reached the also heavily induced level of the transgenic cell lines (Figure S1).

### 2.2. Fast Induction of pr-10a Expression Regulators Sebf and pti4

**Figure 2:**
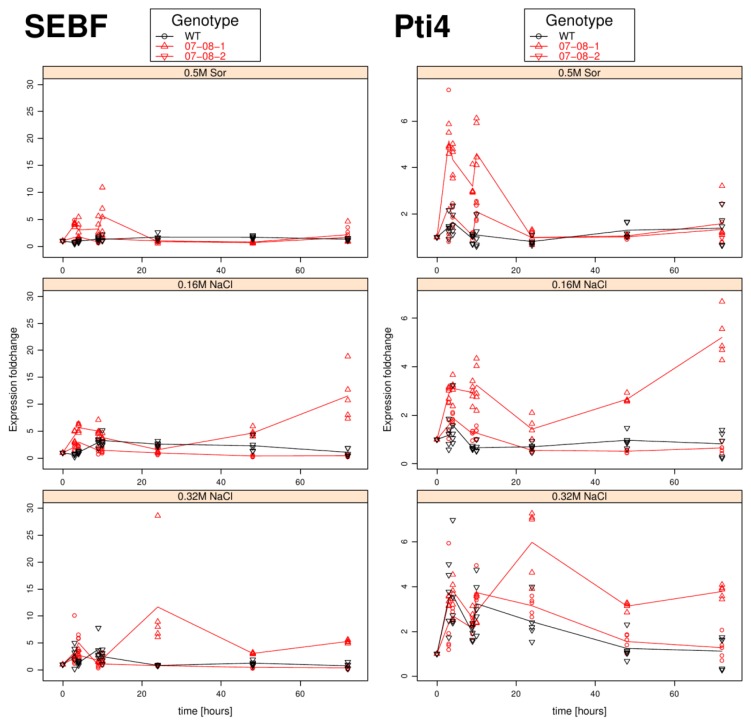
Time course of *sebf* (left column) and *pti4* (right column) gene-expression fold changes of cells osmotically stressed, as indicated. Measurements for all five independent samples per time point and treatment group are displayed as symbols (circles and triangles) indicating individual measurements and lines are giving their averages. WT, wild type.

In analogy to the *pr-10a* expression monitoring transcriptional levels of *sebf* and *pti4*, whose gene products are engaged in the heterodimeric SEBF-Pti4 repressosome complex [22], they were tracked in the same experimental setup and displayed in Figure 2. The according relative expression levels of *sebf* and *pti4* (normalized against *18S rDNA* [47] are provided in Figures S2 and S3).

Interestingly, in contrast to the *pr-10a* expression, the regulator coding genes show a far stronger induction in both transgenic lines compared to the wild type cells (Figure 2). Certainly, both the regulator coding genes showed expression patterns corresponding to that exhibited by *pr-10a* shown above, but mainly differing in the magnitude of the fold changes.

On the 0.5 M sorbitol treatment, the wild type cells exhibited nearly no expression change. The transgenic lines revealed no consistent pattern: Whereas 07-08-2 showed almost no reaction to the treatment when treated with the 0.5 M sorbitol, the transgenic cell lines revealed a slight induction; genotype 07-08-1 reacted with up to five-fold higher gene expression of *pti4* in the first 10 hours.

Under the mild 0.16 M NaCl regime, a slight expression induction during the very first phase of stress treatment, followed by decline to the non-induced level, was revealed only for genotype 07-08-1, whereas both 07-08-2 and the wild type cells did not react with substantial gene expression induction. Further, the comparably weak induction of *pr-10a* expression genotype 07-08-1 exhibited at the 72 h time point (around tenfold see Figure 1B) corresponded to an induction of both *sebf* and *pti4* at that time point (Figure 2).

During the first 10 hours the 0.32 M NaCl treatment generated regulator-expression induction comparable to that from the mild 0.16 M NaCl treatment. However, with increased incubation time, 07-08-1 showed persistently enhanced *sebf* gene-expression induction, as well as nearly no decline in *pti4* expression fold-change. In contrast, wild-type cells and genotype 07-08-2 exhibited nearly equally low expression fold-change values throughout the observation period.

### 2.3. Mannopine-synthase Promoter is Induced by Strong Salt Treatments

**Figure 3:**
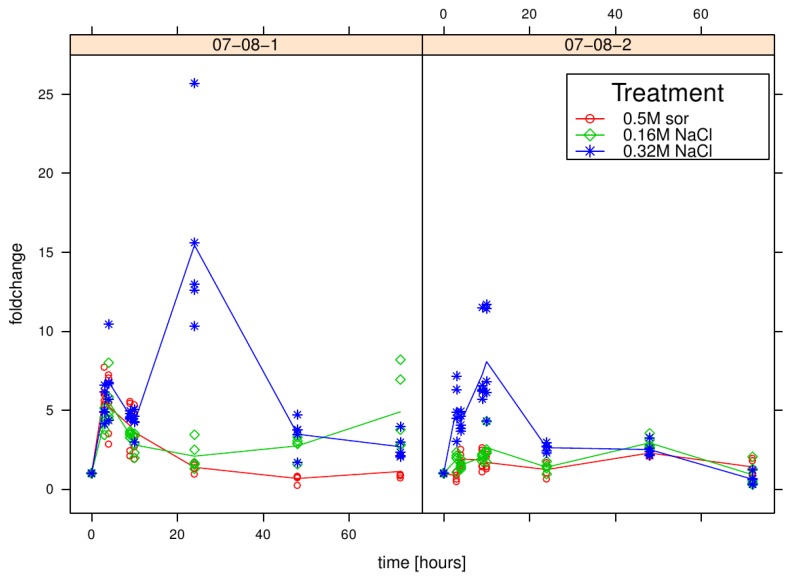
Time course of *luc* gene-expression fold-changes in the transgenic cell lines under osmotic regimes, as indicated. Measurements for all five independent samples per time point and treatment group are displayed as circles, rhombs and asterisks (for 0.5 M sorbitol, 0.16 M NaCl and 0.32 M NaCl supplement in the medium respectively) indicating individual measurements per treatment, while lines give their group means.

Correspondingly to the above described experimental setup, the expression of luciferase, the reporter-gen of the transgenic construct, was monitored. As shown in Figure 3, the luciferase expression clearly increases due to the severe 0.32 M NaCl treatment, with even higher fold changes (about 15-fold) in genotype 07-08-1 compared to an about 8-fold gene induction in genotype 07-08-2. Interestingly, 07-08-1 also reacts to both the mild 0.16 M NaCl and the 0.5 M sorbitol treatment with about seven- to ten-fold *luc* gene expression induction. Genotype 07-08-2 does not exhibit appreciable expression induction under these treatments.

Furthermore, the pattern of luciferase induction is remarkably similar to that observed for *sebf* and *pti4* (Figure 2).

### 2.4. Growth Behaviour Is Influenced by both the Chemical Compound and the Strength of the Osmotic Pressure

**Figure 4:**
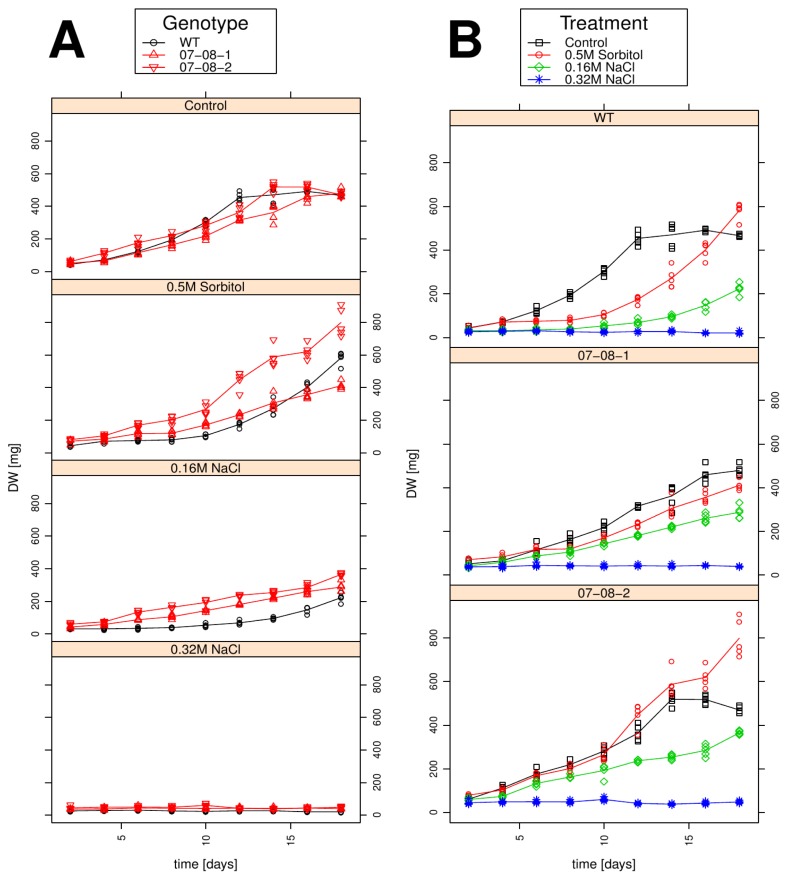
Dry-weight accumulation of wild-type (WT) and transgenic cultures (07-08-1 and 07-08-2) under control conditions and osmotic regimes, as indicated. Dry-weight-measurements for all five independent samples per time point and treatment group are displayed as symbols, indicating individual measurements, while lines give the respective group means. (**A**) Data are assigned to colours and symbols according to the genotypes; (**B**) Data are assigned to colours and symbols according to the treatments.

To phenotypically describe the treatments’ effects on long-term scale, osmotic and salt-stress responses were determined by measurement of cells’ growth behaviour over 18 days. Enabling comprehensive insights into the growth behaviour, the data are presented as both grouped according to treatments (Figure 4A) and grouped according to individual genotypes (Figure 4B).

Under control conditions, growth in all three genotypes starts from the beginning onwards, with very similar growth behaviours (Figure 4A). The 0.5 M sorbitol treatment delayed the onset of growth of wild type cells by approximately 10 days, but then an exponential dry-weight increase until a mean of around 600 mg was reached after 18 days (Figure 4A). Whereas until day 10 both genetically engineered cell lines outplay the wild type cell lines on 0.5 M sorbitol, and since they have shorter delay in gaining dry weight, only 07-08-2 at day 18 is about 200 mg superior to the wild type’s dry weight, while 07-08-1 drops after day 10 below the level of the wild type dry-weight ending up with about 200 mg less at day 18 (Figure 4A).

Also, at the milder salt stress of 0.16 M NaCl, all three genotypes showed growth retardation in comparison to the control condition. The wild type showed a delay of about 12 days before gaining dry-weight, albeit only weakly, until a mean dry-weight of 200 mg was reached after 18 days (Figure 4A). Contrastingly, both transgenic cell lines started growth after four to six days, showed a faster increase in dry-weight accumulation, and at day 18, both reached mean dry weights superior to the wild type’s dry weight.

The 0.32 M NaCl treatment, though being equiosmolal to the 0.5 M sorbitol, apparently caused the largest stress effect, since none of the investigated genotypes showed any growth (Figure 4A).

For all genotypes, growth is more severely retarded by any applied NaCl treatments than by the 0.5 M sorbitol treatment (Figure 4B).

The growth-behaviour of the wild type culture is characterized by a strong sensitivity to mild salt conditions (0.16 M NaCl), and in contrast to the two transgenic genotypes, also by a substantial difference in growth characteristics between control conditions and sorbitol. Interestingly, both transgenic cell lines showed less growth retardation from all three types of osmotic treatments than the wild type (Figure 4B, middle and lower panel). Besides these common patterns, all three genotypes nevertheless showed different response types to the osmotic treatment. In genotype 07-08-01, growth at control conditions 0.5 M sorbitol and 0.16 M NaCl is somewhat similar in the sense that the beginning of growth is quite early (~day 3–5), and that growth is rather linear, irrespective of slight differences in the steepness of growth increase at different treatments. In contrast, though the growth behaviours of 07-08-02 are similar to 07-08-01 in the first 10 days across all four growth conditions, genotype 07-08-02 shows a growth increase under control conditions, and also under 0.5 M sorbitol, from day 10 onwards.

### 2.5. Cell Respiration Reacts on both the Chemical Compound and the Strength of Osmotic Pressure

**Figure 5:**
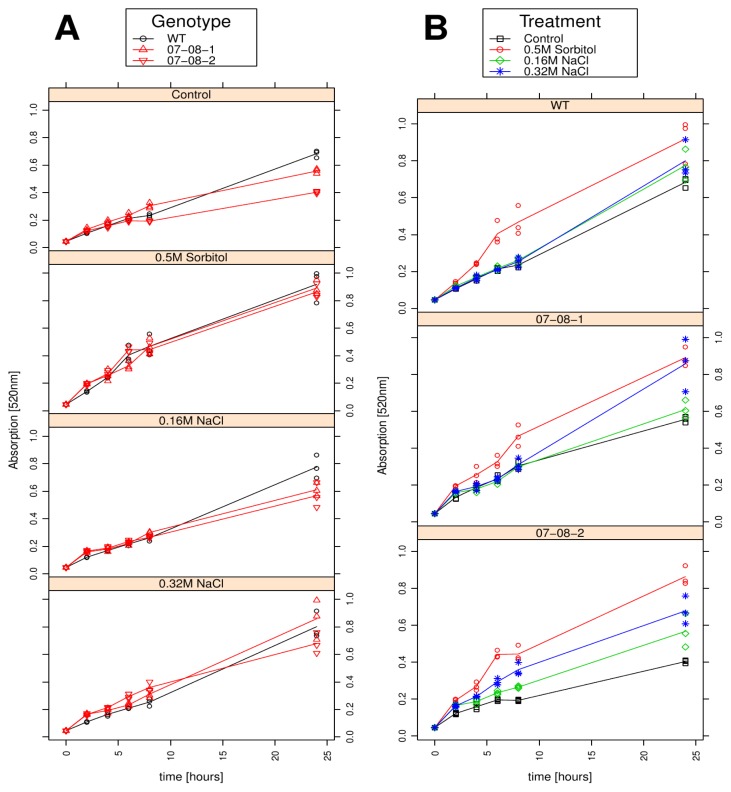
Respiration kinetics of wild-type (WT) and transgenic cultures under control conditions and osmotic regimes as indicated. Measurements for all three independent samples per time point and treatment group are displayed as symbols (circles and triangles) indicating individual measurements, and lines give the averages. (**A**) Data are assigned to colours and symbols according to the genotypes. (**B**) Data are assigned to colours and symbols according to the treatments.

Like the growth curves, the respiration kinetics are presented as both grouped according to treatments (Figure 5A) and grouped according to genotypes (Figure 5B).

Under all investigated conditions, active respiration could be detected from the beginning of the experiment onwards, even though the first measurement was performed only after 2 h (Figure 5A). Also, under all investigated conditions, the respiration kinetics were astonishingly parallel for both the wild type and the transgenic cell lines, particularly for the 0.5 M sorbitol treatment (Figure 5A). After 24 h, the transgenic cell lines accumulated detectably lower formazan intensities under control and the mild 0.16 M NaCl treatment (Figure 5A).

The grouping according to genotypes revealed another interesting feature (Figure 5B): Within each genotype, the respiration kinetics in the first 6–8 h for the control condition and the two salt treatments were highly similar. In contrast, from the beginning onwards, the 0.5 M sorbitol treatment caused a substantially larger increase in formazan accumulation in all three genotypes (Figure 5B). Only at advanced time points, after 24 h, all three genotypes differed in their respiration kinetics at the distinct osmotic treatments (Figure 5B). After 24 h the wild type similarly showed a slightly increased respiration due to the salt treatment, while 07-08-1 reacted strongly to the severe 0.32 M NaCl, but only very slightly to the mild 0.16 M NaCl treatment. In contrast, 07-08-2 reacted sensitively to the applied osmotic pressure and type of compound, exhibiting individual respiration curves for each investigated treatment (Figure 5B).

### 2.6. Wild-Type Cells Showed Sustained Respiration Capacity under Severe 0.32M NaCl

To determine the cell survival under severe salt stress (0.32 M NaCl), the cells’ ability to reduce the formazan into colour was tested after 4 h, 8 h, 24 h, 6 days, 8 days, 10 days and 12 days of this treatment.

**Figure 6:**
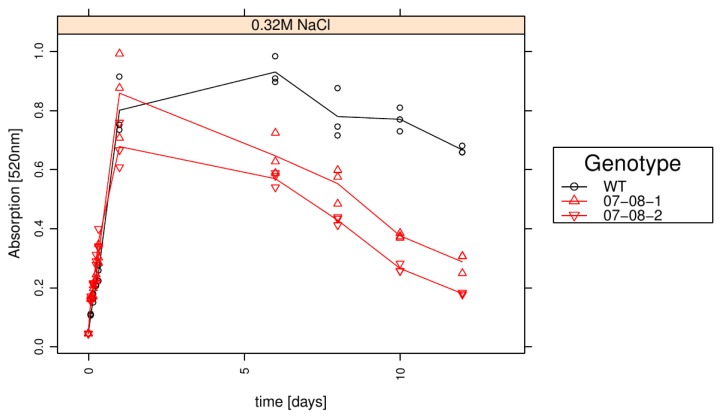
Respiration as measurement of cell survival: Wild-type (WT) and transgenic cultures under 0.32M NaCl treatment in liquid culture were supplemented with Biolog Redox Dye A^®^ after 4 h, 8 h, 24 h, 6 days, 8 days, 10 days and 12 days. The individual absorption measurements for all three Erlenmeyer flasks per genotype and time point (circles and triangles) and the averages (lines) are indicated.

Although the 0.32 mM NaCl condition completely impeded growth in all genotypes (Figure 3), the determination of respiration capacity surprisingly revealed sustained respiration capacities in the wild-type cells, while both transgenic cell lines exhibited a relatively fast decline (Figure 6). Thereby, the respiration performance of transgenic line 07-08-2 remained continually inferior to both that of genotype 07-08-1 and the wild type cells.

## 3. Discussion

Using a set of heterotrophic potato cell lines (wild type and *pr-10a* overexpressing), this study provides an in-depth and comprehensive longitudinal investigation of *pr-10a*-expression patterns and associates this information with newly derived knowledge about the short- and the long-term phenotypes, the resulting respiration behaviour and growth patterns, respectively. To the best of our knowledge, this is the first study providing such detailed information about longitudinal expression patterns of *pr-10a* accompanied with analyses of *sebf* and *pti4*, for a set of both osmotic- and ionic-stressors and in combination with phenotypic information from different levels.

### 3.1. pr-10a Expression Induction by a Common Stimulus

We were able to show that the magnitude of pr-10a expression induction is sensitive to both the chemical compound and the strength of the applied osmotic pressure, but remains highly controlled in terms of the timing of expression induction when it is overexpressed.

Sorbitol, a sugar-alcohol, indeed changes the osmotic pressure, but is not likely to alter the ionic regime of the medium [48,49]. Although it was shown to leak into cells to some extent [50], it does not negatively affect metabolic activity since it does not damage protein hydration. Indeed, [50] the use of sorbitol for the *in vitro* induction of water stress when working with intact *in vitro* potato plants is recommended. But when dealing with dedifferentiated cells, the specific features of the applied compounds affect the cells directly. Possible beneficial effects of sorbitol used as an additional carbon source, as described by [44], are discussed below.

In contrast, NaCl as a ionic, membrane-permeable substance causes ionic stress in the cytoplasma and has severe impacts on protein hydration when attaining the cytoplasma in excess [1,2]. Nevertheless, both sorbitol and NaCl induced the *pr-10a* expression in all investigated genotypes in such a similar pattern that a common stimulus for its gene expression has to be postulated (Figure 1) and that the *pr-10a* overexpression does not change the internal gene-expression regulation (see also below).

### 3.2. Interaction between Treatments and Transcriptional Activity of the Transgene

Forming a heterodimeric SEBF-Pti4 complex, the two proteins were shown to disable the expression of *pr-10a* via the occupation of the silencer element of the promoter [22]. For the activation of *pr-10a* gene expression, the complex has to dissociate from the DNA, and the proteins are hypothesized to also dissociate from each other [22].

Although various details about the transcriptional and posttranscriptional regulation of Pti4 [51–53] and the determining features of the SEBF protein [23,25,54] are known, the data at hand do not permit an unambiguous interpretation. We confirmed that in both transgenic genotypes the relative gene-expression levels revealed a generally higher amount of *pr-10a* transcripts (see Figure S1), indicating a well working, constant overexpression of *pr-10a*. Considering that both cell lines overexpressing *pr-10a* responded only slightly to the applied stresses (Figure 1), the impact of the, apparently still acting, endogenous *pr-10a* genes is supposed to be marginal compared to the total of the *pr-10a* transcripts.

Nevertheless, the comparably high *pr-10a* induction in the transgenic cell lines under the mild salt stress suggests an interaction with the transgenic construct, namely a possible induction effect of the salt treatment onto the mannopine-synthase promoter (p-MAS) [55,56] controlling the transgenic construct [57]. Because the endogenous *pr-10a* responds with high gene-expression induction to the high osmotic-pressure treatments, the enhanced expression of the transgene is not detectable, but under the mild 0.16 M NaCl treatment this expression enhancement is not covered. However, it can be concluded that the *pr-10a* overexpression does not change the internal gene-expression regulation in this early phase of stress response.

Interestingly, with this salinity-dependent induction of the *p-MAS* promoter, the regulators *sebf* and *pti4* also exhibited a well-structured expression pattern. Since the wild-type cells altered neither the *sebf* nor *pti4* gene expression appreciably under the sorbitol or the equiosmolal 0.32 M NaCl treatments, it is supposed that the expression induction in the transgenic lines is an effect of the transgenes.

However, considering the findings of [58] indicating that, among others *pti* genes, *pti4* activates the expression of a wide array of pathogenesis-related genes and plays important and distinct roles in plant-defence mechanisms, it has to be argued that due to unknown feedback, as well as forward-loop regulations, the *pr-10a* overexpression disturbed the adjustment of plant-defence gene regulation by modifying the *pti4* and *sebf* expression levels.

### 3.3. Importance of Phenotypes Derived from Cellular Level

Compared with intact plants, which are able to decelerate or modify the impact of stress conditions by complex interactions between distinct organs and tissues [1,2], no such superordinate mechanisms against salt and/or drought stress are hampering experimental access when using cell cultures.

In the chosen approach here, cells are equally surrounded by the medium, and thus, experience equal conditions at any time during treatment. This facilitates the control of stress homogeneity and the characterisation of cell behaviour independent of influences from plant morphology. This made it possible to distinguish in detail the overlapping effects of ionic and non-ionic solutes supplementing the media (Figure 1). However, compared to whole plants, heterotrophic dedifferentiated cell cultures have only a limited repertoire of phenotypes.

Beside the established, relatively slowly developing growth curves, a feature measurement for short-term cell responses appears essential. Thus, a method for monitoring the cell respiration behaviour using a modified cell-viability assay was adopted in this work. Here, mitochondrial respiration leading to the production of Nicotinamide adenine dinucleotide **(**NADH) engenders a redox potential and a flow of electrons to reduce a tetrazolium dye (TTC) [36], thereby producing insoluble purple-coloured formazan. The more rapid the cellular respiration (*i.e.*, the electron flow), the faster purple colour is formed [37,38]. TTC-based viability assays in plant cells utilize the difference between the colourless solution of TTC in water and the insoluble red formazan accumulating inside intact, living cells with a functional mitochondrial electron-transport chain [39]. In plant sciences, traditional applications of this assay are mainly aimed at only qualitatively determining the viability of cells (dead versus alive). To this end, the accumulated formazan is extracted from the cells and its quantity is photometrically determined.

The underlying assumption is that living cells produce the red formazan, which accumulates in the cells, while dead cells lose their membrane integrity, causing the formazan to escape into the surrounding medium [40].

Various studies suggested that quantitative evaluations are also feasible [40–42] and that additional information could be generated from quantifying both the finally accumulated amount of formazan as well as the amount during the course of its formation, *i.e.*, the course of respiration over time [43,44].

Since growth is initially inhibited, but later on enhanced, together with primarily enhanced respiration, by the sorbitol treatment, it can be hypothesized that this sugar alcohol stresses the cells osmotically from the beginning onwards, but with sustained treatment, cells can make use of sorbitol as a source of energy. Similar phenomena were observed by [44] who stressed heterotrophic *Zea mays* L. cell cultures with sorbitol treatments. Metabolomic analyses indicated a fundamental adjustment of the carbon-cycling apparatus [59] suggesting that, after cells have adapted to the new osmotic regime, sorbitol is used simply as an additional carbon source. Carbon balance as a major integrator of plants’ response to stress [60], and thus the maintained ATP production throughout stress, is seen as the crucial point to facilitate tolerance mechanisms [61]. Nevertheless, the primary osmotic stress phase has to be managed, and at least transgenic line 07-08-2 seems to take advantage from the *pr-10a* overexpression in this early phase, as this line is able to adapt more quickly (Figure 3).

### 3.4. Technical Aspects of Respiration Measurements in Plant Cell Cultures

The underlying assumption for the respiration measurements is that electrons flow through the mitochondrial respiratory electron chain, which causes the formazan formation, automatically leading to ATP synthesis [38,46]. Since the plant mitochondrial respirator chain can be by-passed using the non-energy conserving alternative oxidase pathway (reviewed in [62]), it has been hypothesized that the formed purple colour in TTC based viability assays must not automatically correspond to ATP production rates. However, experimental findings concerning the correlation of ATP production and formazan-based respiration assays are ambiguous [42,44,63], and thus, more research on this issue is required.

To enable a similarly highly automated approach for measuring plant cell respiration behavior, as it was introduced for microbial respiration [38], some challenges have to be mastered. Due to the bigger cell size of plant cells, the authors do not see a straightforward application using the automated Biolog Phenotype MicroArray™ technique [46], which is based on 96-well plates. Plant cells, even from suspended cell cultures, are not suitable to be pipetted in volumes of 100 μL, and inoculation of wells adjusted according to turbidity measurements is also hardly feasible.

Additionally, with the TTC derivative used here, the purple-coloured formazan accumulates inside the cells, which makes the procedure of respiration measurements over time cost- and labour-intensive, since extraction of formazan has to be accomplished. However, for mammalian cells, TTC derivatives are available, whose formazan product remains solubilized and membrane-permeable (B. Bochner, Biolog Inc., pers. comm). Thus, further research should aim at the discovery of tetrazolium derivatives, or formulations that can escape plant cells after colour formation, and thus, simplify the process of measurement by superseding the ethanol-extraction step.

## 4. Experimental Section

### 4.1. Plant Material and Osmotic Challenge

As shown by [57], dicistronic constructs achieve the coordinated co-expression of a physically independent target protein, here the *pr-10a*, providing a physiological trait, along with firefly luciferase as reporter protein. A non-embryogenic suspension culture of *Solanum tuberosum* cv. Désirée (DSMZ No. PC-1182), and two independently derived transgenic cell lines constitutively homologously overexpressing *pr-10a* (07-08-1 and 07-08-2, see [19], were sub-cultured weekly by transferring 40 mL of suspension to 60 mL of fresh 4× medium [64] containing 2 mg L^−1^ 2,4-dichlorophenoxyacetic acid (2,4 D), 0.5 mg L^−1^ indole-3-acetic acid (IAA), 0.5 mg L^−1^ 1-naphtylacetic acid (NAA) and 0.4 mg L^−1^ kinetin, pH 5.6 and incubated in a 300 mL Erlenmeyer flask on a gyratory shaker (TR-250, Infors AG, Basel, Switzerland) with 50 mm orbit (100 rpm) at 25 °C.

To yield preculture cells for the separate experimental approaches (see below), cells were harvested from the logarithmic growth phase three days after subculturing. To this end, cell mass was filtered off the medium through a Nylon net (100 μm pore size, NeoLab, Heidelberg, Germany) using a Buchner funnel.

To discriminate between ionic and non-ionic effects, a non-ionic osmotic challenge (0.5 M sorbitol) and a ionic equiosmolal sodium-chloride (0.32 M NaCl) treatment were chosen, both representing the four-fold (800 Os/kg) osmotic pressure of the control treatment (200 Os/kg). The treatment set was further augmented by a milder 0.16 M NaCl treatment (resulting in 500 Os/kg) producing about 2.5 fold osmotic pressure compared to control treatment. Compared with the control 4× medium (200 mOs/kg) the mild salt treatments (0.16 M NaCl) increased the osmotic pressure by a factor of 2.5, whereas the high salt and the sorbitol treatments lead to an increase by a factor of 4.

### 4.2. RNA Isolation and Quantitative Real-Time PCR

An inoculum of 15 g preculture cell material (see above) was transferred into a sterile 300 mL Erlenmeyer flask and filled up to 100 mL with 4× medium supplemented with either 0.16 M NaCl or 0.5 M sorbitol or 0.32 M NaCl.

Cell material was sampled after 3 h, 4 h, 9 h, 10 h, 24 h, 48 h and 72 h incubation time. Medium was removed by filtering off the cells through a Nylon net (100 μm pore size, NeoLab, Heidelberg, Germany) in a Buchner funnel. For each time-point and treatment, five independent flasks were inoculated and harvested independently, resulting in 425 individual samples. After harvesting, the cell mass was immediately frozen in liquid nitrogen and stored therein until further analysis.

The frozen cell material was ground under liquid nitrogen using sterilised mortar and pistil. Total RNA was extracted from 100 mg fresh weight ground material with TriFast Gold (Peqlab, Erlangen, Germany) according to the manufacturer’s instructions. cDNA-synthesis was carried out with the RevertAid First Strand Synthesis kit (Fermentas, St. Leon Roth, Germany). For relative quantification of the *pr-10a* and luciferase mRNA by PCR, TaqMan probes against the *pr-10a* sequence (primers pr-10a129(f) 5′-TACACATGAAGCCACAAGCA-3′, pr-10a129(r) 5′-ATGCTTCCATCTCCC TCAGT-3′, probe: pr-10a129 5′-TCAAAGCTTTGGTTGTTGATGCTGA-3′) and the *luc* sequence (primers luc141(f) 5′-TATGAACATTTCGCAGCCTA-3′, luc141(r) 5′-ATCGACTGAAAT CCCTGGTA-3′, probe: luc141 5′-GTTTCCAAAAAGGGGTTGCAAA-3′) were used.

For the relative quantification of the *sebf* and *pti4-*mRNA by PCR TaqMan probes (primers; (f) 5′-CCTTCTCCAATGGCTTCTTC-3′, SEBF(r) 5′-GTTGTTTGGGAAGTGGGTTT-3′, probe: SEBF (famtam) 5′-TCCCTCCATTTCCTTTCACTTACACCA-3′ and Pti4(f) 5′-GGTTCAATGAAA CGGAGAAGA-3′, Pti4(r) 5′-GGACACCTGTCAATTGTTCG-3′, Probe: Pti4(famtam) 5′-CCGT CACATTTCCGAACGGC-3′) were used.

As internal standard, primers against the 18S rRNA gene 18S138 (f) 5′-TAAAGGAATTGACGGAAGGG-3′, 18S138 (r) 5′-CACCACCACCCATAGAATCA-3′, probe: 18S138 5′-CGCAGGCTCCACTCCTGGTG-3′ were used according [65]. Quantitative real-time PCR was performed on an Eppendorf Mastercycler ep realplex4 platform using the following program: 30 s 95 °C; (40× (95 °C, 5 s; 60 °C, 20 s)).

To determine the effect of the osmotic challenges for each genotype separately, cells treated with the 4× medium served as the corresponding control group. In Figure S1, the time course of the corresponding relative expression levels of *pr-10a* (normalized against 18S [47] are given. Considering that smaller ΔCt values indicate higher expression levels, both transgenic cell lines showed substantially higher *pr-10a* expression levels from the beginning onwards.

### 4.3. Dry-Weight Determination of Cell Material

For testing stress tolerance by growth, an inoculum of 1g of preculture cells (see above) per 100 mL Erlenmeyer flasks was filled up with 50 mL of 4× medium supplemented with either 0.16 M NaCl or 0.5 M sorbitol or 0.32 M NaCl. The flasks were sealed with aluminum foil and incubated on a gyratory shaker (TR-250, Infors AG, Basel, Switzerland) with 50 mm orbit (100 rpm) at 25 °C for up to 18 days.

Cell material was sampled every two days where five flasks were harvested independently for each treatment and dry weight was determined. To this end, the fresh cell material was transferred quantitatively into pre-weighed Petri dishes and dried to constant weight at 60 °C for 72 h. Petri dishes including dry cell matter were weighted again after drying. This experiment was repeated three times. Since no differences between these experimental repetitions were found, for matters of convenience, the data sets of only one repetition have been presented.

### 4.4. Respiration Curves

For the respiration measurements, the 100 mL Erlenmeyer flasks were set up analogously to the growth experiment with each 50 mL medium additionally supplemented with 500 μL of Biolog Redox Dye A^®^ (100×) (Biolog, Hayward, CA, USA). The flasks were sealed with aluminium foil and incubated on a gyratory shaker (TR-250, Infors AG, Basel, Switzerland) with 50 mm orbit (100 rpm) at 25 °C for up to eight hours in the dark. The formazan development was recorded after 2 h, 4 h, 6 h, 8 h and 24 h. For each treatment-genotype combination three flasks were set up and harvested independently. The flasks’ content was transferred to 50 mL PP tubes (Cellstar^®^ Greiner bio-one, Frickenhausen, Germany), and cell material was collected by centrifugation with 4200× *g* for 10 min. Supernatant was discarded. To stop the respiration activity of the cells, the pellet was immediately frozen at −80 °C [40]. For extraction of the tetrazolium dye, the pellets were resuspended in 4 mL ethanol (technical grade) and incubated in the dark over night at room temperature according to [43]. By centrifugation (15 min at 18,000× *g*) cell debris was removed and the absorption of the supernatant was determined photometrically at 520 nm (TECAN Infinite M200 with 96-well flat bottom Greiner bio-one PS Microplates). This experiment was repeated three times. For matters of convenience, datasets of only one experimental repetition have been presented.

As recommended by [40], the validity of the testing system was ascertained as follows: Possible interactions between the used dye and the applied media were investigated; the correlation between formazan development in actively growing cells and samples killed by freezing was also determined. Furthermore, tests on background absorption due to extractable cellular pigments were performed, and the absorption spectra of the ethanol extracts were validated (see Figure S4). Neither an interaction between the dye and the applied medium itself, nor false-positive formazan development in the here used *Solanum tuberosum* cv. Désirée cell cultures was observed.

### 4.5. Assessment of Cell Survival via Respiration Measurements

An inoculum of 1 g preculture cells (see above) per flask was filled up with 50 mL of 4× medium supplemented with 0.32 M NaCl. The flasks were sealed with aluminum foil and incubated on a gyratory shaker (TR-250, Infors AG, Basel, Switzerland) with 50 mm orbit (100 rpm) at 25 °C. After 4 h, 8 h, 24 h, 6 d, 8 d, 10 d and 12 d to each three flasks per genotype 500 μL of Biolog Redox Dye A^®^ (100×) (Biolog, Hayward, CA, USA) were added and incubated for additional 24 h. The amount of formed formazan was determined as described above.

### 4.6. Statistical Analysis

Calculation of both dCT and ddCT values was done using spreadsheets and the statistical software R [66]. Values were calculated as fold change 2^−ΔΔct^ for *pr-10a* standardized to the *18S rDNA* threshold cycle (ΔCt) with the differences between the treated cells and cells from control group. A fold change of 1 indicates no change in *pr-10a* expression caused by the treatment [47].

For graphical representation of experimental results, basic plotting functions from the statistical software R [66] together with the add-on package lattice [67] were used.

For the evaluation of the growth curves, a cell means model was set up describing the dry weight in dependency of the treatment (dry weight~treatment), whereas the treatment levels specify the combination of the genotype, the applied medium and the time of measurement, resulting in 108 levels. Analogously, for evaluation of respiration curve results, a cell-means model was set up, describing the absorption in dependency on the treatments (absorption~treatment), whereas the resulting 72 treatment levels consist specify the combination of the genotype, the applied medium and the time of measurement.

For comparisons of experimental group means simultaneous multiple-comparison procedures according [68] were performed with R [66] using the above-mentioned linear cell-means models.

## 5. Conclusions

For the data at hand, it can be concluded that the *pr-10a* expression is (i) induced by both the osmotic and ionic stress, that (ii) *pr-10a* expression induction is highly controlled over time and the *pr-10a* overexpression does not change the internal gene-expression regulation in early phases of stress response. Moreover, (iii) the overexpression, at least for the severe 0.32 M NaCl stress investigated here, is not exclusively beneficial, and thus, provides insights about the impact of the artificially enhanced presence of *pr-10a* on cell physiology.

This study strongly supports that respiration measurements can provide valuable information, especially during the very first phase of stress exposure, *i.e.*, a short period of time in which the determination of growth reactions is usually infeasible. Furthermore, this technique is likely to facilitate studies on the metabolic activities of cells under even severe stress conditions or other adverse circumstances [48], as we have also shown here that respiration can occur independently of cell growth [35,69].

With the expected improvement of measurement techniques, the longitudinal character of the output data will become more important. Data complexity will increase and contain additional information coded in the shape characteristics of the curves [70]. As it was shown for microbial organisms [71], these curve features can unravel fundamental differences or similarities in the respiration behaviours under distinct treatments, which may be identifiable only by curve comparisons rather than by the simpler comparisons of means. Software solutions for analyses regarding different experimental questions are already freely available [72] and (http://cran.r-project.org/web/packages/opm/index.html).

However, once adapted to a mid- to high-throughput measurement approach, this type of data comprise the same information content as those known from bacterial phenotyping, and thus, have comparable potential for testing gene functions and improving genome annotation in the plant cell system, too.

## Supplementary Materials

### 1. Monitoring of Gene-Expression

The monitoring of gene-expression was accomplished by treating the suspended cell cultures with osmotic (0.5 M sorbitol) and salt stress (0.16 M and 0.32 M NaCl) followed by (q)PCR-based expression measurement over time, as described in detail in the experimental section. In Figures S1–S3 the according relative expression levels of *pr-10a*, *sebf* and *pti4* (normalized against *18S rDNA* [47], respectively) are provided.

### 2. Validation of TTC-Based Respiration Curves

To validate the outcome of TTC-based respiration measurements, tests on background absorption, due to, for *e.g.,* extractable pigments, and analysis of the resulting absorption spectra were performed according to recommendations by [40]. Accordingly, wild type cells were grown under control medium for 24 h. The absorption spectra of corresponding ethanol extracts were measured and compared to spectra from ethanol-extracts from samples, where wild type cells have been incubated 8 h and 24 h in control medium supplemented with Biolog Dye A (Figure S4).

The ethanol extracts from cells grown under control conditions did not result in accountable absorption values (Figure S4), indicating no disturbing cell compounds could be extracted. Cells provided with Biolog Dye A produced the red formazan and the corresponding spectra exhibited a narrow peak with maximum at 520 nm (green line in Figure S4). Thus 520 nm was chosen for all absorption measurements determining formazan amounts.

Further possible interactions between the used dye, the applied media and compounds of deceased cells were investigated (Figure S5). Therefore, cell material was inactivated by freezing and thawing, and incubated 24 h with the different media (see Experimental Section) either supplemented with Biolog Dye A or without any further supplement. Formazan formation was determined by absorption-measurement of corresponding ethanol extracts at 520 nm. As positive-controls ethanol-extracts from wild type cells grown under 0.5 M sorbitol supplemented with Biolog Dye A for 2 h and 24 h were chosen.

Neither an interaction between the dye and the applied media itself, nor false positive formazan formation caused by cell debris in the here used *Solanum tuberosum* cv. Desirée cell cultures, was observed (Figure S5A,B).

Figure S1 Results of relative *pr-10a* expression analysis of cells treated with differently supplemented medium (control, 0.5 M sorbitol, 0.32 M NaCl, 0.16 M NaCl). Given are the expressions levels standardized to their 18S rRNA threshold cycle (ΔCt). Wild type (WT) is encoded by black lines and circles, transgenic cell cultures (07-08-1 and 07-08-2, respectively) by red lines and triangles, while symbols indicate single measurement points and lines corresponding group means. Note that smaller ΔCt values indicate a stronger expression [1].

Figure S2 Results of relative sebf expression analysis of cells treated with differently supplemented medium (control, 0.5 M sorbitol, 0.32 M NaCl, 0.16 M NaCl). Given are the expressions levels standardized to their 18S rRNA threshold cycle (ΔCt). Wild type (WT) is encoded by black lines and circles, transgenic cell cultures (07-08-1 and 07-08-2, respectively) by red lines and triangles, while symbols indicate single measurement points and lines corresponding group means. Note that smaller ΔCt values indicate a stronger expression [1].

Figure S3 Results of relative pti4 expression analysis of cells treated with differently supplemented medium (control, 0.5 M sorbitol, 0.32 M NaCl, 0.16 M NaCl). Given are the expressions levels standardized to their 18S rRNA threshold cycle (ΔCt). Wild type (WT) is encoded by black lines and circles, transgenic cell cultures (07-08-1 and 07-08-2, respectively) by red lines and triangles, while symbols indicate single measurement points and lines corresponding group means. Note that smaller ΔCt values indicate a stronger expression [47].

Figure S4 Comparison of absorption spectra of ethanol extracts from potato wild type cells on control medium (black circles), against ethanol extracts from wild type cells treated with 4X medium supplemented with Biolog Dye A after eight (red circles, positive control) and 24 h (red crosses, positive control 2). The green dashed line indicates the absorption maximum at 520 nm.

Figure S5 Absorption of ethanol extracts derived from inactivated cell material incubated 24 h in differently supplemented media (see Experimental Section) plus Biolog Dye A. Given are mean and standard deviation from three independent replicates. Wild type cells (WT) are indicated by light grey bars, genetically engineered cell lines (07-08-1 and 07-08-2 respectively) are indicated by dark grey bars. (**A**) Absorption at 520 nm of ethanol extracts from differentially treated cells without Biolog Dye A supplement; (**B**) Absorption at 520 nm of ethanol extracts from inactivated cells supplemented with Biolog Dye A.
