# Supplementary figures and images for: Impacts of pr-10a Overexpression at the Molecular and the Phenotypic Level
Source: Int J Mol Sci. 2013 Jul 22;14(7):15141–66. doi: 10.3390/ijms140715141 (PMC3742292; doi:10.3390/ijms140715141)

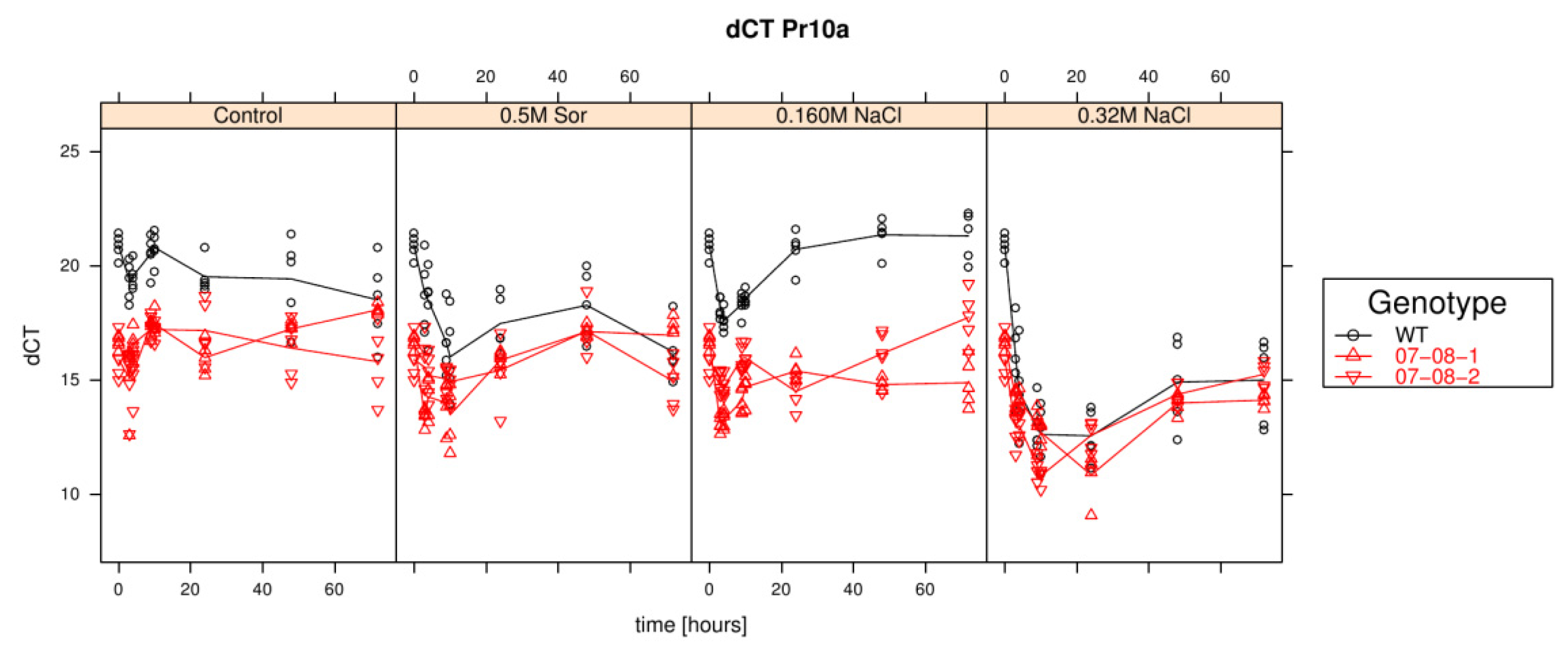

Supplement: Figure S1 — Results of relative pr-10a expression analysis of cells treated with differently supplemented medium (control, 0.5 M sorbitol, 0.32 M NaCl, 0.16 M NaCl). Given are the expressions levels standardized to their 18S rRNA threshold cycle (ΔCt). Wild type (WT) is encoded by black lines and circles, transgenic cell cultures (07-08-1 and 07-08-2, respectively) by red lines and triangles, while symbols indicate single measurement points and lines corresponding group means. Note that smaller ΔCt values indicate a stronger expression [1]. [file ijms-14-15141s1.tif]

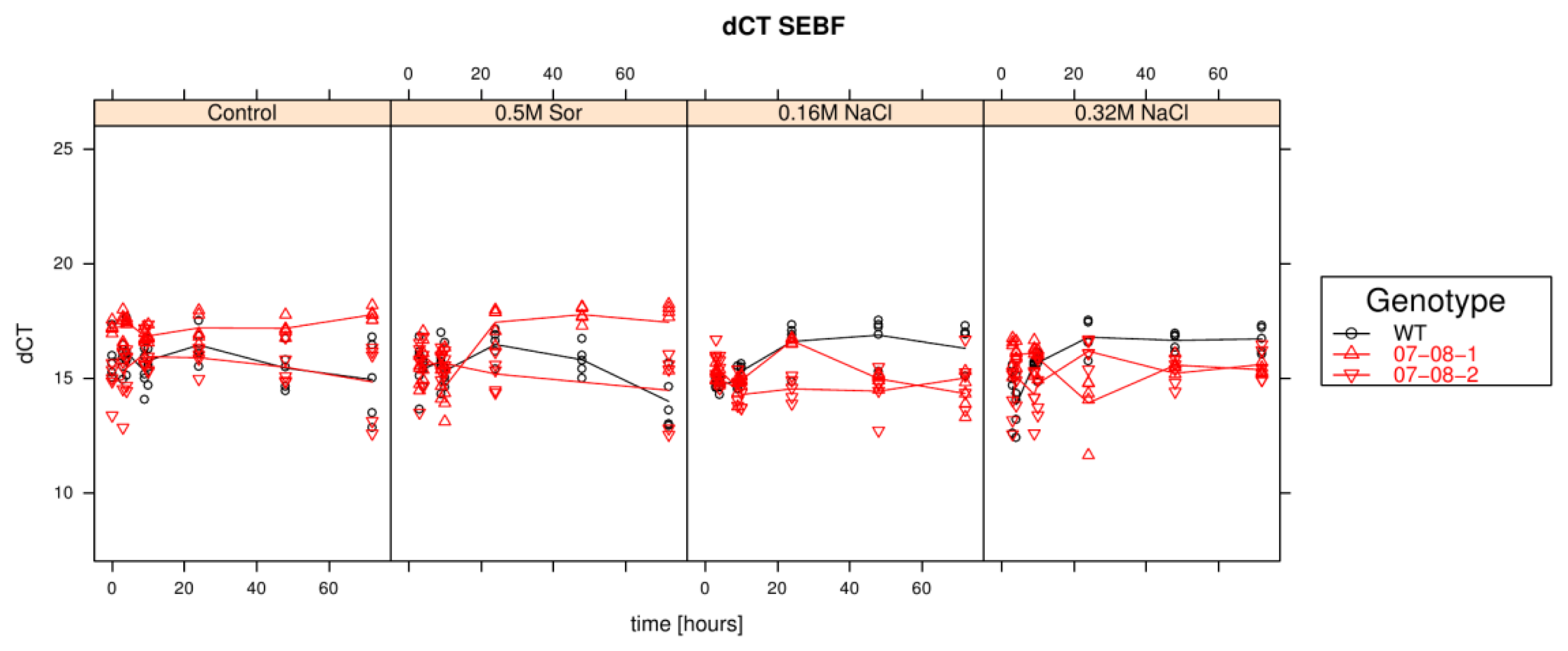

Supplement: Figure S2 — Results of relative sebf expression analysis of cells treated with differently supplemented medium (control, 0.5 M sorbitol, 0.32 M NaCl, 0.16 M NaCl). Given are the expressions levels standardized to their 18S rRNA threshold cycle (ΔCt). Wild type (WT) is encoded by black lines and circles, transgenic cell cultures (07-08-1 and 07-08-2, respectively) by red lines and triangles, while symbols indicate single measurement points and lines corresponding group means. Note that smaller ΔCt values indicate a stronger expression [1]. [file ijms-14-15141s2.tif]

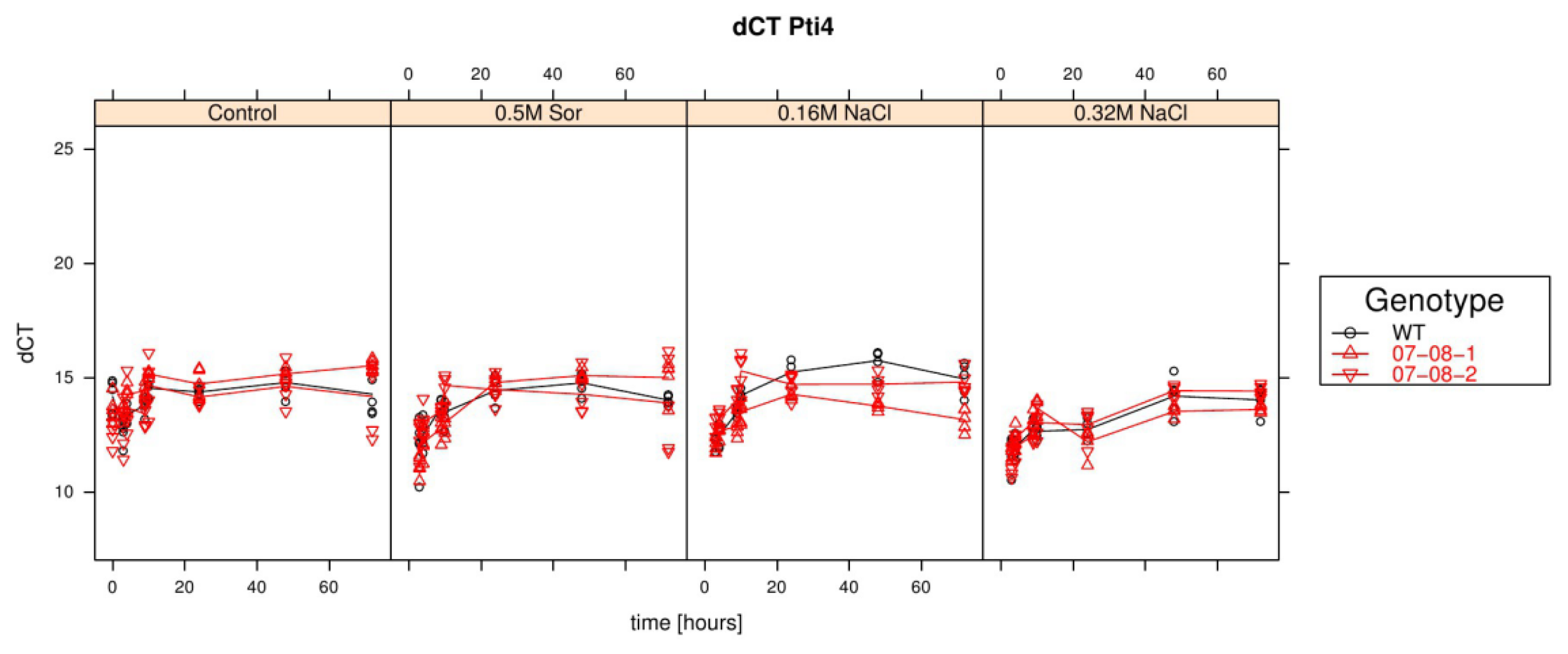

Supplement: Figure S3 — Results of relative pti4 expression analysis of cells treated with differently supplemented medium (control, 0.5 M sorbitol, 0.32 M NaCl, 0.16 M NaCl). Given are the expressions levels standardized to their 18S rRNA threshold cycle (ΔCt). Wild type (WT) is encoded by black lines and circles, transgenic cell cultures (07-08-1 and 07-08-2, respectively) by red lines and triangles, while symbols indicate single measurement points and lines corresponding group means. Note that smaller ΔCt values indicate a stronger expression [47]. [file ijms-14-15141s3.tif]

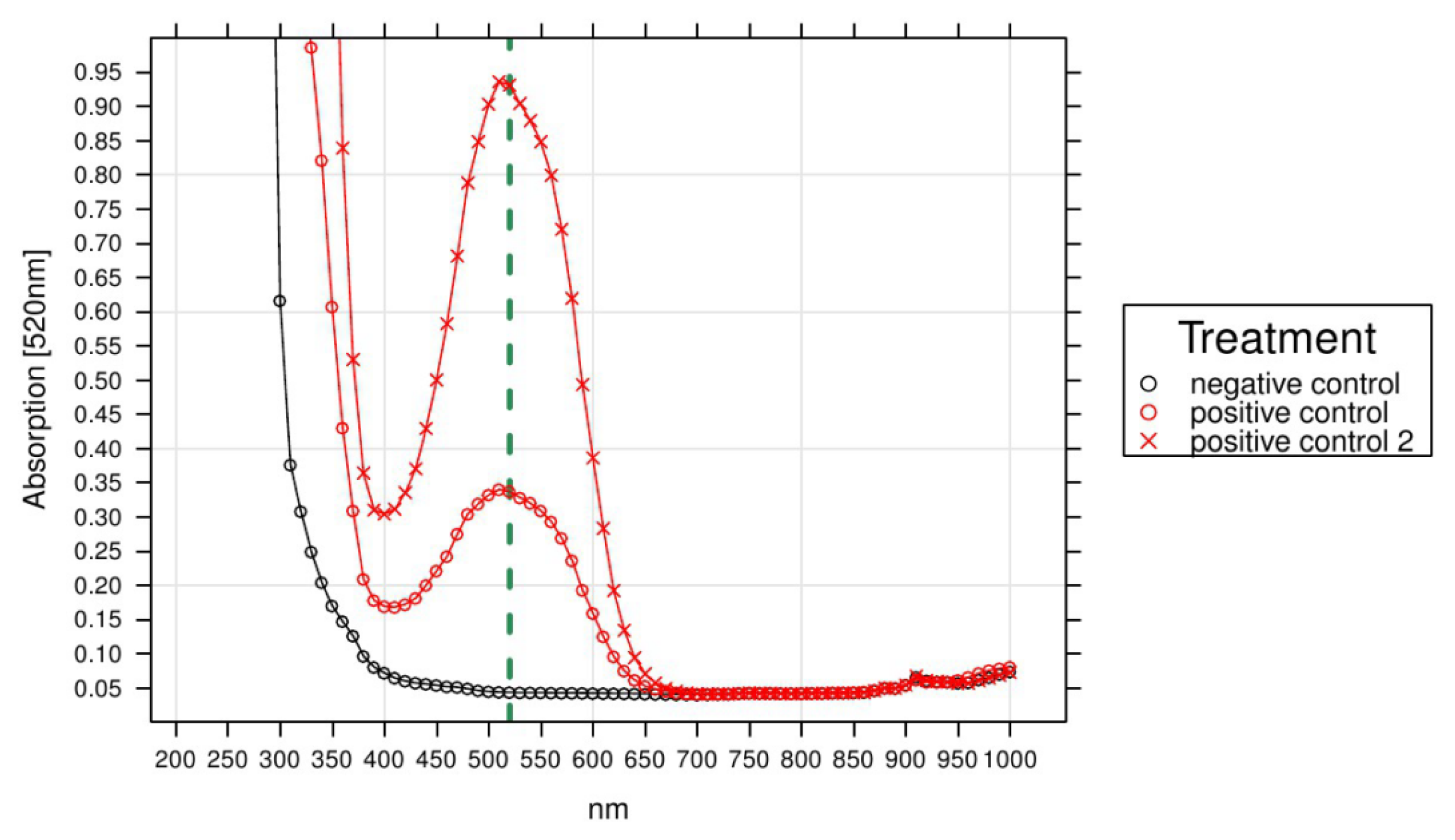

Supplement: Figure S4 — Comparison of absorption spectra of ethanol extracts from potato wild type cells on control medium (black circles), against ethanol extracts from wild type cells treated with 4X medium supplemented with Biolog Dye A after eight (red circles, positive control) and 24 h (red crosses, positive control 2). The green dashed line indicates the absorption maximum at 520 nm. [file ijms-14-15141s4.tif]

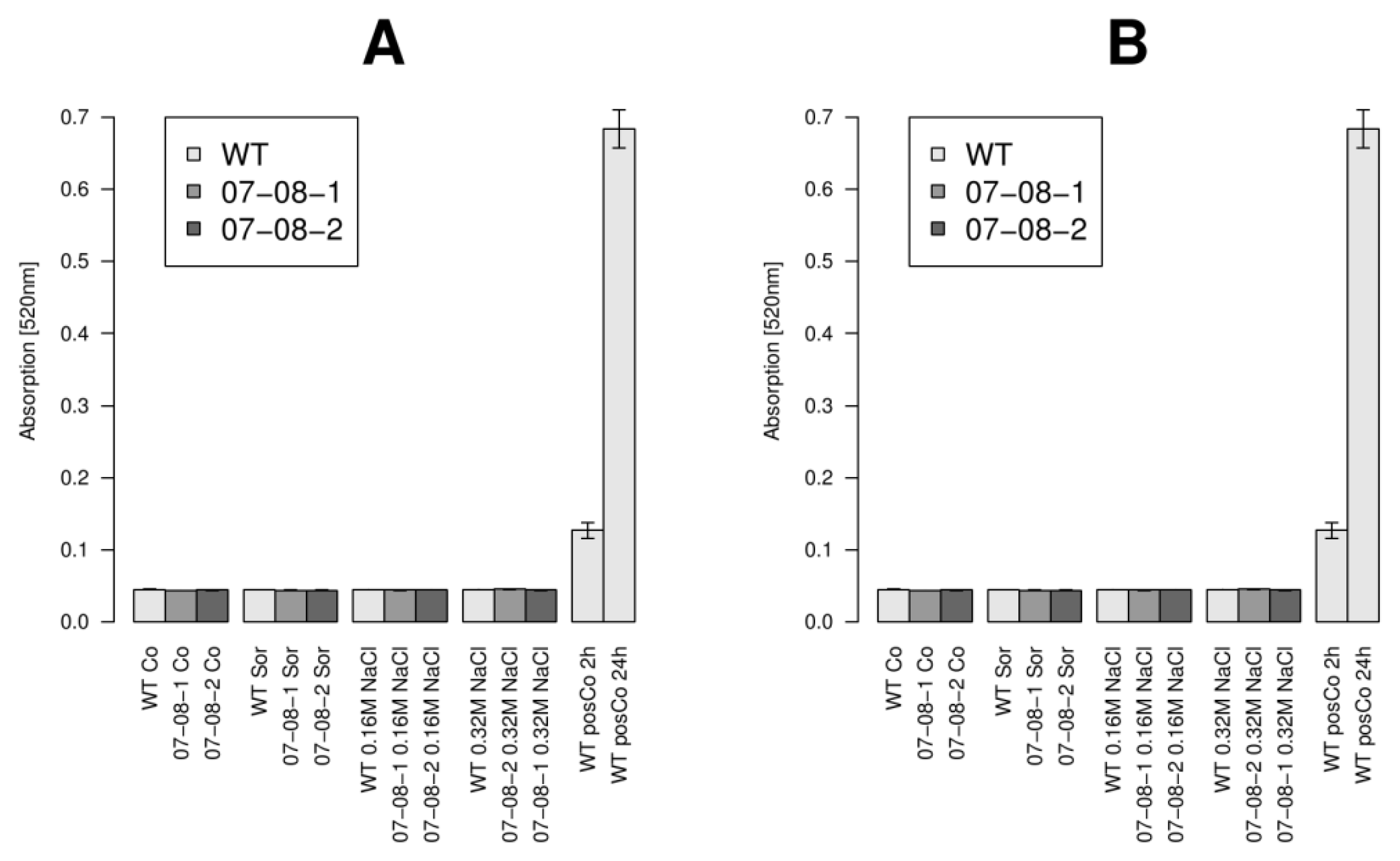

Supplement: Figure S5 — Absorption of ethanol extracts derived from inactivated cell material incubated 24 h in differently supplemented media (see Experimental Section) plus Biolog Dye A. Given are mean and standard deviation from three independent replicates. Wild type cells (WT) are indicated by light grey bars, genetically engineered cell lines (07-08-1 and 07-08-2 respectively) are indicated by dark grey bars. (A) Absorption at 520 nm of ethanol extracts from differentially treated cells without Biolog Dye A supplement; (B) Absorption at 520 nm of ethanol extracts from inactivated cells supplemented with Biolog Dye A. [file ijms-14-15141s5.tif]
